# Anthropometric Measurements of the Human Distal Femur: A Study of the Adult Malay Population

**DOI:** 10.1155/2013/175056

**Published:** 2013-11-05

**Authors:** Fitdriyah Hussain, Mohammed Rafiq Abdul Kadir, Ahmad Hafiz Zulkifly, Azlin Sa'at, Azian Abd. Aziz, Md. Golam Hossain, T. Kamarul, Ardiyansyah Syahrom

**Affiliations:** ^1^Medical Devices & Technology Group (MEDITEG), Universiti Teknologi Malaysia, 81310 Johor Bahru, Johor, Malaysia; ^2^Department of Orthopaedics, Traumatology and Rehabilitation, Kulliyyah of Medicine, International Islamic University Malaysia, 25200 Kuantan, Pahang, Malaysia; ^3^Department of Radiology, Kulliyyah of Medicine, International Islamic University Malaysia, 25200 Kuantan, Pahang, Malaysia; ^4^Tissue Engineering Group, NOCERAL, Department of Orthopaedic Surgery, Faculty of Medicine, University of Malaya, 50603 Kuala Lumpur, Malaysia; ^5^Sport Innovation and Technology Centre, Universiti Teknologi Malaysia, Skudai, 81310 Johor Bahru, Johor, Malaysia

## Abstract

The distal femurs of 100 subjects (50 men, 50 women) from the Malay population aged between 19 and 38 years were scanned to measure the anterior-posterior (AP) and medial-lateral (ML) width. The mean AP values were 64.02 ± 3.38 mm and 57.33 ± 3.26 mm for men and women, respectively, and the mean ML values were 74.91 ± 3.52 mm and 64.53 ± 3.07 mm. We compared our data to that published previously for the Chinese and Indian populations. It was found that the Malay population had smaller distal femur than that of the Chinese but was larger than that of the Indian population (*P* < 0.05). In conclusion, although it is well established that Asians have a smaller distal femur size than that of the Western population, the variations in different Asian ethnicities may need to be considered when designing the appropriate knee implant.

## 1. Introduction

Joint replacement involving the distal femur requires the use of highly complex surgical techniques, as this would involve the accurate placement of well-fitted implants and adequate balancing of the surrounding soft tissues [1, 2]. The use of an appropriate femoral component size is essential to maintain the normal functional range of motion of the knee. In addition, a mismatch between the prosthesis size and bone may result in a number of severe complications [3–5]. It has been demonstrated that using an undersized component will result in implant loosening, whilst an oversize component may cause impingement of the surrounding soft tissues. The use of appropriate component size is therefore crucial to produce long-term success following total knee arthroplasty (TKA) [6–10]. 

Most arthroplasty of the knee are normally performed on the diseased knees to return the knee to its normal physiological function. Most of these pathological diseases of the joint affected people at the age of 45 and above. There were, however, cases where total knee replacements were performed on the young age group of 45 and below for treatment of rheumatoid arthritis, juvenile rheumatoid arthritis, and osteoarthritis [11, 12]. Follow-up studies reported cases of posttraumatic arthritis, avascular necrosis, hemochromatosis, lupus, dislocation, sepsis, unstable components, and osteolysis for this age group.

It is well known that the Asian population has a smaller distal femoral and proximal tibia size than that of its Western counterpart [13–15]. Due to the comparatively smaller built and stature of the Asian population, many surgeons believe that imported implants, which are mainly designed from the morphometrics gained from the Western population, may not be suitable for patients located in Asian countries [15]. It is very likely that in most surgeries an oversized component would have been used in many Asian centers, resulting in poor implant performance [16]. It is therefore of paramount importance that an appropriate femoral size for the different demographical and ethnic populations be used for the appropriate individuals. This would ensure that the implants use would provide an optimal performance during its lifetime.

Anthropometric measurements of the knee joint from many Asian countries are presently available. These include countries like China [14], India [15], Japan [17], Korea [18], Taiwan [16], and Thailand [19]. However, the measurements of the majority of the population located in many Southeast Asian countries, for example, Malaysia, Singapore, Indonesia, and so forth, which consist mainly of the Malay population, have not been previously described in any known literatures.

The aim of the current study is therefore to determine the anthropometric measures of the distal femur amongst the young adult Malay population in Malaysia, measured using three-dimensional models reconstructed from computer tomographic (CT) images datasets. The final derived data will then be compared with different ethnic anthropometric data reported in other literatures.

## 2. Materials and Method

### 2.1. Study Overview

The approval to conduct this study was obtained from the Ethical Committee from the Clinical Research Centre (CRC), Hospital Tengku Ampuan Afzan (HTAA), Kuantan, Malaysia. One hundred healthy individuals were randomly recruited for this study. Inclusion and exclusion criteria were set prior to subject selection, which excludes subjects who have a known history of trauma to the lower limb or congenital abnormalities. Subjects were asked to sign an informed consent prior to the study. Subjects were later administered with a standard questionnaire identifies various demographic data, for example, age, sex, and so forth. Fifty men and women, respectively (*n* = 50 each), were recruited for the study, all aged between 19 and 38 years (mean 22.71 ± 3.70).

### 2.2. Methods

**Figure 1:**
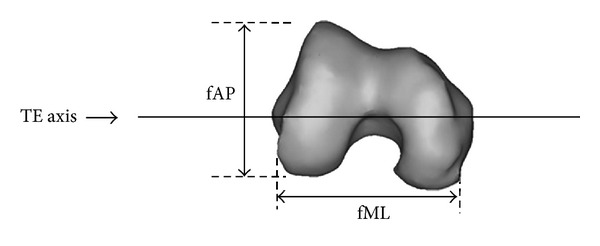
The mediolateral and anteroposterior measurements taken from the reconstructed femur model.

A four-row multislice CT scanner (Somatom, Volume Zoom, SIEMENS) set with fixed scanning parameters of 3 mm slice thickness, recon increment of 1.5 mm, collimation of 1.25 mm, 12 mm table feed per rotation, 90 mAs and 120 kV were used in this study. The scan was conducted to include images from the iliac crest to the ankle. During the scanning procedure, the foot was placed within a custom-designed foot jig to standardize the position and angulations of the lower limbs. All subjects were properly shielded from the ionizing radiation using the standard and recommended gonadal shields. No contrast media were used in this study. Three-dimensional (3D) models of the knee joint were reconstructed using the raw data obtained from the scanned images obtained through the use of AMIRA 4.0 software. The anteroposterior and mediolateral measurements were performed through the use of the analysis tool SolidWorks 2009 as shown in Figure 1.

### 2.3. Data Comparison

To compare the data obtained in this study to that of previously published data, various databases which included Pubmed, Scopus, ISI, and Google were searched for keywords which include but not limited to “anthropometry,” “knee,” “total knee arthroplasty,” “China,” and “India.” China and India were focused on in this study due to their relevance in geometric location. In addition, Chinese and Indian descents are also considered as a local population in many Southeast Asian countries.

There were 461 papers published in this area. After excluding similarities, cross publications, and irrelevance to the subject matter, only 3 papers were deemed appropriate for this study. Two studies were related to the Chinese population while only 1 was available for the Indian population. Data for these studies were extracted from the published papers and extrapolated for statistical analyses.

### 2.4. Statistical Analysis

The independent *t*-test was used to find the differences between males and females in femoral measurements and body dimensions. To examine the average relationship between femoral measurements (AP and ML) and age and body dimensions, multiple regression analysis was applied. The underlying multiple linear regression model corresponding to each variable is

(1) $$\begin{array}{c} Y=\beta_{0}+\beta_{1}X_{1}+\beta_{2}X_{2}+\beta_{3}X_{3}+\varepsilon , \end{array}$$

where *X*_1_ (age), *X*_2_ (body height), and *X*_3_ (BMI) are the predictor variables (independent variables) and *Y* is the response variable (AP and ML). *β*_0_ is the intercept term, *β*_1_, *β*_2_, and *β*_3_ are the unknown regression coefficients of age, body height, and BMI, respectively, and *ε* is the error term with *N*(0, *σ* ^2^) distribution. An important assumption of multiple regression analysis is that the predictor variables are independent of each other. However, in some applications of regression, the predictor variables were related to each other, creating a multicollinearity problem. A variance inflation factor (VIF) was used in this study to check for the multicollinearity problem among the predictor variables. The variance inflation for independent variables *X*_*j*_ is

(2) $$\begin{array}{c} {\text{VIF}}_{}j=\frac{1}{1-R_{j}^{2}},\;j=1,2,3, \end{array}$$

where *R*_*j*_^2^ is the square of the multiple correlation coefficient of the *j*th variable with the remaining variables, where

1. if 0 < VIF < 5, there is no evidence of a multicollinearity problem;
2. if 5 ≤ VIF ≤ 10, there is a moderate multicollinearity problem;
3. if VIF > 10, there is serious multicollinearity problem of variables [20]. Finally, *t*-test was used to find the differences between two populations. Statistical significance was accepted at *P* < 0.05 and the analyses were carried out using SPSS software version 15.

## 3. Results

The anthropometric measurements of the knees are summarized in Table 1. The average values for AP measures were 63.94 ± 3.40 and 57.40 ± 3.36 for men and women, respectively, while the average values for the ML measures were 74.86 ± 3.62 and 64.53 ± 3.21 for men and women, respectively.

Prior to statistical analysis, the collected data were tested for normality to determine their suitability for use in the parametric test. The normality of AP and ML was determined using Kolmogorov-Smirnov normality test. There was no problem concerning the normality of data distribution for the AP and ML measures for both sexes, as the *P* values were greater than 0.05 (Table 2).

### 3.1. Demographic Measurements of the Malay Population

The present study also measured various demographic parameters within the Malay population. This was done in order to determine whether the demographic changes can influence the femoral size of any individual. The mean ages of the males and females were 23.88 ± 4.52 and 21.54 ± 2.12 years, respectively. The mean height, weight, and BMI of Malay males were 170.96 ± 6.37 cm, 70.76 ± 14.38 kg, and 24.40 ± 4.68 kg/m^2^, respectively, while for women the average height, weight, and BMI were 156.02 ± 6.17 cm, 53.31 ± 13.11 kg, and 21.98 kg/m^2^, respectively. The average mean values observed for AP and ML for males and females were 64.02 ± 3.38 mm and 74.91 ± 3.52 mm, and 57.33 ± 3.26 mm and 64.53 ± 3.07 mm, respectively. The observed values of AP and ML for males were significantly (*P* < 0.01) higher than that of females (Table 3).

Pearson correlation coefficient analysis demonstrated that the antero-posterior (AP) diameter was highly positively correlated with the medio-lateral (ML) diameter for males (*r* = 0.623, *P* < 0.001) and females (*r* = 0.684, *P* < 0.001).

### 3.2. Comparison between the Malay Populations and Other Populations Based on Published Data

The data from the study which consisted mainly of the Malay population were compared with different Asian ethnic populations, which are China and India. There were no significant differences between the ML measures in men for the Malay and Chinese populations. All other comparisons demonstrated significant differences as shown in Table 4. An overall comparison between the different races demonstrates that the Chinese have the largest AP and ML measurements followed by the Malay and Indian populations (Table 5).

## 4. Discussion

An important factor required to achieve long-term success in total knee arthroplasty surgeries is the use of geometrical matched prosthesis, which simulates the natural conditions of knee joints. In addition, these prostheses are required to adequately cover the exposed bony surfaces, thereby providing good gliding motions during knee flexion or extension. In order to achieve this, the use of an appropriately sized implant would be mandatory. Moreover, the implant design must incorporate the appropriate AP and ML measures of the normal knee. It has been described in many studies that to determine the morphometric measurements of the normal knees, datasets need to be obtained from scanned CT images analyzed from the knees of a normal population [13, 14, 21]. Many manufacturers design their implants using data acquired through this method; however, these data were mainly based on Western populations [16, 18, 21]. Although there have been many previous reports demonstrating that Asians have smaller knees than that of the Western population [13–15, 22], data comparing the different ethnicities within the various Asian nations appear to be lacking. It can be argued that the differences in knee measurements, if present within these groups, would not pose a significant influence in the clinical outcome; however, studies to refute or support these claims appeared to be absent. What can be ascertain at this juncture is the fact that implant companies designing total knee arthroplasty components have made great strides in adopting the measurements of the Asian population into newer implant designs, specifically targeting to market the product to these populations. This indicates the reality of the conditions and how important it is for the appropriate implant size to be made available for an intended population [13].

Significant differences in the anthropometric measurements of the knee between the different ethnic groups were observed in this study. This is an important finding given the fact that Asians have been generally regarded to be of the same size [18]. It identifies the mediolateral (ML) diameter and anteroposterior (AP) measurement of the knee in detail with methods corresponding to that previously described [13, 14]. The benefit of doing this is twofold: it allows the comparison to be made between the data collected from different studies to that of ours and the comparison of these data to match presently available implant sizes. The choice to compare the present data to two major ethnicities in Asia, that is, Chinese and Indian, is prudent considering that these populations are those which have been considered by many implant designers as the normal population representing the populations of this region. In addition, being a multiethnic nation composed of 3 major ethnicities which includes the Chinese and Indian descents, the study on the Malay population is appropriate as this would also provide a good comparison of the different ethnicities.

The distal femurs for females are generally smaller than those of males, with marked narrowing of the distal femurs clearly observed. These findings were also observed in other studies which compare the different ethnic groups [13, 15]. The data obtained also underwent further analyses to determine the correlations between the various anthropometric measures of the individuals. It is clear that strong correlations can be found between the measured ML and AP dimensions with height and BMI, thus suggesting that a larger implant size is necessary to adequately fit a larger individual. In the present progressive society, this appears to be important as it has been shown that a rising BMI is apparent in many nations, more so for the Asian population who at present are benefiting from a healthy economic growth for many years now. Data from this study is therefore necessary to provide the latest knee measurements which over time would be applicable for future Asian implant designs.

The purpose of the present study was to establish the normal knee measurements of the Malay population and by doing so, compare it to the published data of the populations located in China and India. In contrast to previous methods, we measured the normal healthy knees instead of resected knees to establish a baseline data for our normal population study [13]. Measurement on resected knees has shown to produce differences in the measured dimensions due to variations in bone resection techniques and the implant used. Furthermore, the use of osteoarthritic knees to model normal conditions may not be wise as bony structural changes leading to deformity changes will produce misleading and misrepresented data [23].

One limitation of this study was the comparative analysis with older age groups from the Chinese and Indian ethnic data. Though there are cases of knee replacement on younger age groups, our thorough search in the literature found no reports on anthropometry of the young age group [11]. We are at present continuing to collect data of these subset populations as well as the mismatch component sizes of the implants used in many of our patients within this region. These data may prove to be useful for future implant designs that wish to incorporate designs that may be applicable for a larger number of ethnic groups in Asia. This in turn would lead to a lower production cost for total knee arthroplasty implants whilst providing the correct sized implants for many patients.

## 5. Conclusion

In conclusion, the present study suggests that Asian knee sizes cannot be universally applied throughout the Asian population since there are significant differences observed between the different ethnicities within 9 the Asian population itself. Future implant designs may wish to incorporate this finding so as to be able to provide better implant fittings for use in the Asian population at a larger scale.

## Figures and Tables

**Table 1:** Anthropometric measurements of distal femur in Malay ethnic group.

	ML	AP	ML/AP
Malay male	74.88 ± 3.55	63.93 ± 3.36	1.17 ± 0.05
Malay female	64.53 ± 3.12	57.39 ± 3.29	1.13 ± 0.05

Note: AP: anterior-posterior; ML: medial-lateral; ML/AP: ratio.

**Table 2:** Kolmogorov-Smirnov normality test for ML and AP of study populations.

Sex	Variable	Statistic	df	*P* value
Male	ML	0.084	49	0.150
	AP	0.064	49	0.150

Female	ML	0.073	48	0.150
	AP	0.096	48	0.150

Note: AP: anterior-posterior; ML: medial-lateral.

**Table 3:** Descriptive statistics of different demographical parameters and anthropometric measurements of Malay adults.

		*N*	Mean	SD	95% CI for mean		Minimum	Maximum	Sex difference
					Lower	Upper			
Age	Male	49	23.98	4.51	22.60	25.30	20	38	2.33**
	Female	48	21.65	2.10	21.04	22.30	20	32	

Height	Male	49	170.76	6.27	169.00	172.56	153	185	14.72**
	Female	48	156.04	6.30	154.21	157.87	141	170	

Weight	Male	49	70.96	14.46	66.81	75.11	40	110	17.57**
	Female	48	53.39	13.37	49.51	57.27	39	118	

BMI	Male	49	24.30	4.60	23.00	25.62	16	35.8	2.26*
	Female	48	22.04	6.50	20.15	23.93	16.7	58.5	

ML	Male	49	74.88	3.55	73.86	75.90	67.30	83.00	10.35**
	Female	48	64.53	3.12	63.62	65.44	58.00	73.00	

AP	Male	49	63.93	3.36	62.96	64.90	57.50	72.80	6.54**
	Female	48	57.39	3.29	56.43	58.35	50.80	65.00	

ML/AP	Male	49	1.17	0.05	1.16	1.18	1.02	1.27	0.05**
	Female	48	1.12	0.05	1.11	1.13	1.03	1.24	

**1% level of significance and *5% level of significance; *N*: number of subjects; AP: anterior-posterior; ML: medial-lateral; SD: standard deviation.

**Table 4:** Comparison between the Malay population and the Chinese and Indian populations.

Ethnic population	Sex	Response variable	*P* value
Malay versus Indian	Male	ML	0.001
		AP	0.001
	Female	ML	0.001
		AP	0.001

Malay versus Chinese	Male	ML	0.417
		AP	0.001
	Female	ML	0.001
		AP	0.001

**Table 5:** The ML and AP measurements for the Malay, Chinese, and Indian populations.

	ML	AP
Malay male	74.88 ± 3.55	63.93 ± 3.36
Malay female	64.53 ± 3.12	57.39 ± 3.29
Chinese male	74.4 ± 2.9	66.6 ± 2.40
Chinese female	66.8 ± 3.10	61.0 ± 2.70
Indian male	69.64 ± 3.11	61.09 ± 3.74
Indian female	61.06 ± 3.11	54.47 ± 1.91
